# A quasi-experimental approach using telemetry to assess migration-strategy-specific differences in the decision-making processes at stopover

**DOI:** 10.1186/s12898-020-00307-5

**Published:** 2020-07-08

**Authors:** Heiko Schmaljohann, Thomas Klinner

**Affiliations:** 1 Institute for Biology und Environmental Sciences (IBU), Carl von Ossietzky University of Oldenburg, Carl-von-Ossietzky-Straße 9-11, 26129 Oldenburg, Germany; 2 Institute of Avian Research, An der Vogelwarte 21, 26386 Wilhelmshaven, Germany

**Keywords:** Bird, Common redstart, Departure decision, Energy stores, European robin, Migration, Radio-tracking, Stopover, Strategy

## Abstract

**Background:**

Migrant birds travel between their breeding areas and wintering grounds by alternating energetically and physiologically demanding flights with periods of rest and fuelling, so-called stopovers. An important intrinsic factor influencing the decision to resume migration is the amount of energy stores available for the next flight. Correlative studies with free-flying birds and experimental studies with caged birds have shown that the amount of energy stores affects the day-to-day, within-day and the directional decision of departure. The methodological advantages of both the correlative and experimental approach are combined when radio-tagging many individuals on the same day and subsequently determining the departure decisions at a high spatiotemporal resolution. Making use of such a quasi-experimental approach with an automated radio-tracking system at stopover, we studied the effect of energy stores on departure decisions and whether they vary between species of different migration strategies experiencing contrasting time constraints. For this, we chose a long-distance migrant, the common redstart (*Phoenicurus phoenicurus*), and a medium-distance migrant, the European robin (*Erithacus rubecula*), because the former has to travel at relatively higher speed to reach its wintering ground in a reasonable time at the expense of relatively higher energetic costs for travelling than the latter.

**Results:**

Common redstarts with higher energy stores were more likely to resume migration than their conspecifics with lower energy stores, whereas this pattern was absent in the European robins. The amount of energy stores significantly affected the timing of departure within the day, with large energy stores yielding early departures in both species. Departure directions from the stopover site during the first night after capture were oriented towards the seasonally appropriate direction but were not affected by variation in energy stores.

**Conclusions:**

We demonstrate the importance of variation in energy stores on the departure decisions and that it may affect species with different migration strategies dissimilarly in autumn. Nevertheless, knowledge of other intrinsic factors, such as feeding conditions, health status and physiological consequences of previous flights, is additionally required to better understand the departure decisions of migrants, as this is the key to providing an overall assessment of the decision-making process.

## Background

While travelling between breeding areas and wintering grounds, migrant birds alternate their flight bouts with periods of rest, so-called stopovers. Since actively flying during the endurance airborne periods is energetically and physiologically extremely demanding [1–3], birds sleep [4], recover [5, 6] and fuel [7] during a stopover. If birds have recovered and prepared for the next upcoming migratory flight, and if environmental conditions are favourable, these intrinsic and extrinsic conditions signal the decision-making processes of the innate migration program to continue migration [8–10].

Energy stores are limiting the duration and thus the distance of the migratory flight bout [11–15]. Therefore, a key intrinsic condition influencing the decision, whether to leave or to remain at the current stopover site is the currently available amount of energy stores [7]. There is strong correlative evidence from studies with free-flying birds that variation in energy stores affects the departure decision on three levels: (i) night-to-night [16–20], (ii) within the day [18, 21–24], but see also Bolshakov et al. [23] and Bulyuk and Tsevy [25] and (iii) in terms of the direction of the route [17, 21, 22]. Higher energy stores generally advance both temporal decisions and lead to seasonally most appropriate directions especially when encountering an ecological barrier.

To experimentally investigate the causal effect of the variation in energy stores on these three departure decisions, we can make use of two observations of caged nocturnal migrants [26, 27]. First, these birds show spontaneous nocturnal migratory restlessness when caged during the migration periods [28–30] and the amount of restlessness is a good approximation for the population-specific migration distance [31, 32] and the actual departure probability [33] and timing [34] in the wild. Second, this migratory restlessness is generally directed towards the flight direction under free-flying conditions [35, 36] and can be measured in Emlen funnels under controlled conditions [37]. The results of such cage experiments show that the departure decision on the night-to-night level [38–42], within the night [21, 43] and with respect to the direction [44, 45] strongly support the findings of the correlative studies under free-flying conditions (see above). Despite the importance of such experiments, we should, however, be aware that because birds adjust their behaviour in relation to the variation of a certain condition in the lab, this does not necessarily mean that they would show this response also in the wild where other conditions might overwrite the response as expected from the cage experiments.

To combine the advantages of correlative and experimental studies, Goymann et al. [20] performed a quasi-experimental study to assess the importance of energy stores on one of the departure decisions of free-flying garden warblers (*Sylvia borin*) on the Italian Mediterranean Island Ventotene during spring migration. For this, they radio-tagged ten “lean” and “fat” individuals on two consecutive days with similar weather conditions and thus minimized the effect of weather variation on the birds’ departure decision. The “fat” birds resumed migration on the day of capture, whereas the “lean” birds remained on the island for up to several days, demonstrating that departure probability increases with energy stores [20]. Their telemetry system allowed precisely determining the presence or absence of the radio-tagged birds, but it did not provide detailed information about the exact departure timing within the day or on the departure direction.

To fill parts of these gaps, we performed a similar quasi-experimental study with free-flying nocturnal migrant songbirds. We caught actively migrating common redstarts (*Pheonicurus phoenicurus*, redstart hereafter) and European robins (*Erithacus rubecula*, robin hereafter) on the small island (2 km^2^) of Helgoland in the German Bight during autumn. Birds of each species were trapped on a single day and immediately released after radio-tagging. Their departure timing and direction were determined using an automated digital telemetry system covering about 30 km^2^ [21]. Instead of categorizing the birds as “lean” or “fat”, cf. Goymann et al. [20], we estimated the energy stores in relation to the bird’s lean body mass following [46] and thus obtained a continuous variable.

Our first objective was to describe the variation in the species’ energy stores, minimum stopover duration, nocturnal departure timing and departure direction. Our second objective was then to assess whether the departure decisions on the night-to-night level, within the night and with respect to the direction of the route were affected by energy stores. Based on the previous findings (see above), we hypothesized that (i) the departure probability increases with energy stores, (ii) the departure within the night advances with energy stores and (iii) the departure directions of the birds are influenced by their current energy stores.

During autumn, long-distance migrants are supposed to be more time-constrained than medium-distance migrants because the former have to travel at relatively higher migration speed to reach their wintering grounds in a reasonable time at the expense of relatively high energetic costs for travelling [18, 47]. If so, medium-distance migrants could afford travelling slower to minimize energy expenditure of transport [48, 49]. These different time constraints are likely to exert different selection pressures, which may explain the differences in the migration strategies between these two groups of birds [18, 24, 47]. As the redstart is a long-distance migrant wintering south of the Sahara and the robin a medium-distance migrant wintering in North Africa to central Europe [50], our third objective was to assess whether the two species differ in their departure decisions and whether variation in their energy stores affected their decisions differently.

## Results

19 redstarts were caught on the 2nd of September 2018. The weather at sunset was characterized by east north-easterly wind (70°), i.e., blowing towards 250°, with wind speed of 7.5 m/s, no precipitation and air temperature of 19 °C. 21 robins were caught on the 6th of October 2018 experiencing northerly wind (350°), i.e., blowing towards 170°, with wind speed of 6.6 m/s, no precipitation and air temperature of 14 °C at sunset. Five redstarts, for which the minimum stopover duration could not be ascertained, were not considered any further in the analyses. Departure directions were obtained for twelve redstarts and 20 robins.

**Fig. 1:**
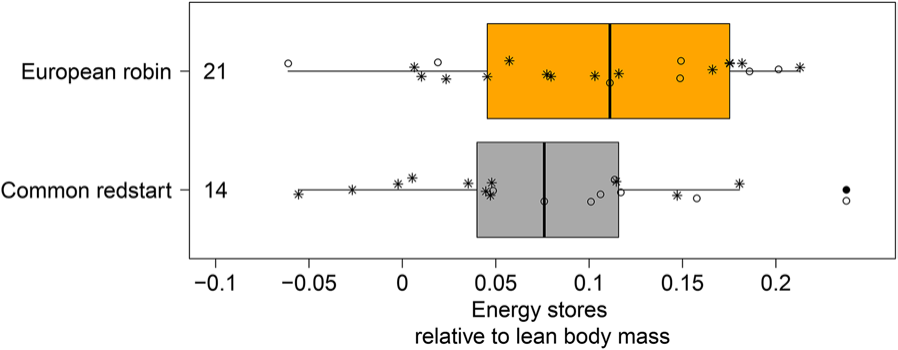
Energy stores of common redstarts (grey) and European robins (orange) at the time of capture. Box plots show the 5th, 25th, 50th, 75th, 95th percentile and one outlier (filled dot). Raw data are presented as open circles indicating individuals departing on the night after capture or stars depicting individuals staying more than 1 day on Helgoland. Numbers on the left side of the box represent sample sizes

Energy stores at capture varied from − 0.05 to 0.2 relative to the individual lean body mass in both species (Fig. 1) and did not differ significantly between them (linear model (LM): n_redstarts_ = 14, n_robins_ = 21, intercept(redstart) = 0.077 ± 0.020 (mean ± standard error (SE)), species(robin) = 0.027 ± 0.026 (mean ± SE), F_1,33_ = 1.1, p = 0.31). We found no effect of the timing of capture within the day on variation in the energy stores in either species (LM_redstart_: n = 14, slope = -0.02 h^−1^ ± 0.01 h^−1^ (mean ± SE), F_1,12_ = 4.3, p = 0.06; LM_robin_: n = 21, slope = -0.01 h^−1^ ± 0.02 h^−1^ (mean ± SE), F_1,21_ = 1.1, p = 0.53).

**Fig. 2:**
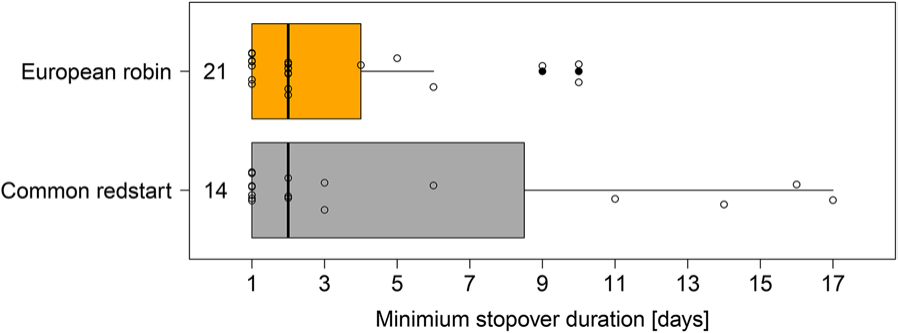
Variation in minimum stopover duration in common redstarts (grey) and European robins (orange) on Helgoland during autumn migration. Box plots show the 5th, 25th, 50th, 75th, 95th percentile and outliers (filled dots). Numbers on the left side of the box represent sample sizes. Raw data are presented as open circles

**Fig. 3:**
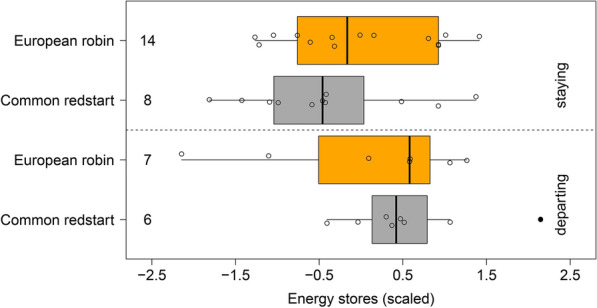
Energy stores (scaled per species) of common redstarts (grey) and European robins (orange) that departed during the first night capture (“departing”, lower panel) and that stayed longer at the stopover site (“staying”, upper panel). Box plots show the 5th, 25th, 50th, 75th and 95th percentile. Numbers on the left side of the box represent sample sizes. Raw data are presented as open circles. See Fig. 2 for the original values of energy stores per species

Minimum stopover duration varied between one and 17 days (Fig. 2) and was not found to differ between the species (Mann–Whitney U test: W = 155.5, p = 0.78). Departure probability during the first night after capture was significantly affected by the amount of energy stores in the redstarts (Mann–Whitney U test: W = 18, p = 0.024), but not in the robins (Mann–Whitney U test: W = 26, p = 0.54; Fig. 3). This indicates that redstarts with higher energy stores were more likely to resume migration than birds with lower energy stores on the day of capture. We found no significant difference in the departure probability between the species (redstarts: 43% [6 out of 14]); robins: 33% [7 out of 21]; Fisher’s exact test: p = 0.72) (Fig. 3).

**Fig. 4:**
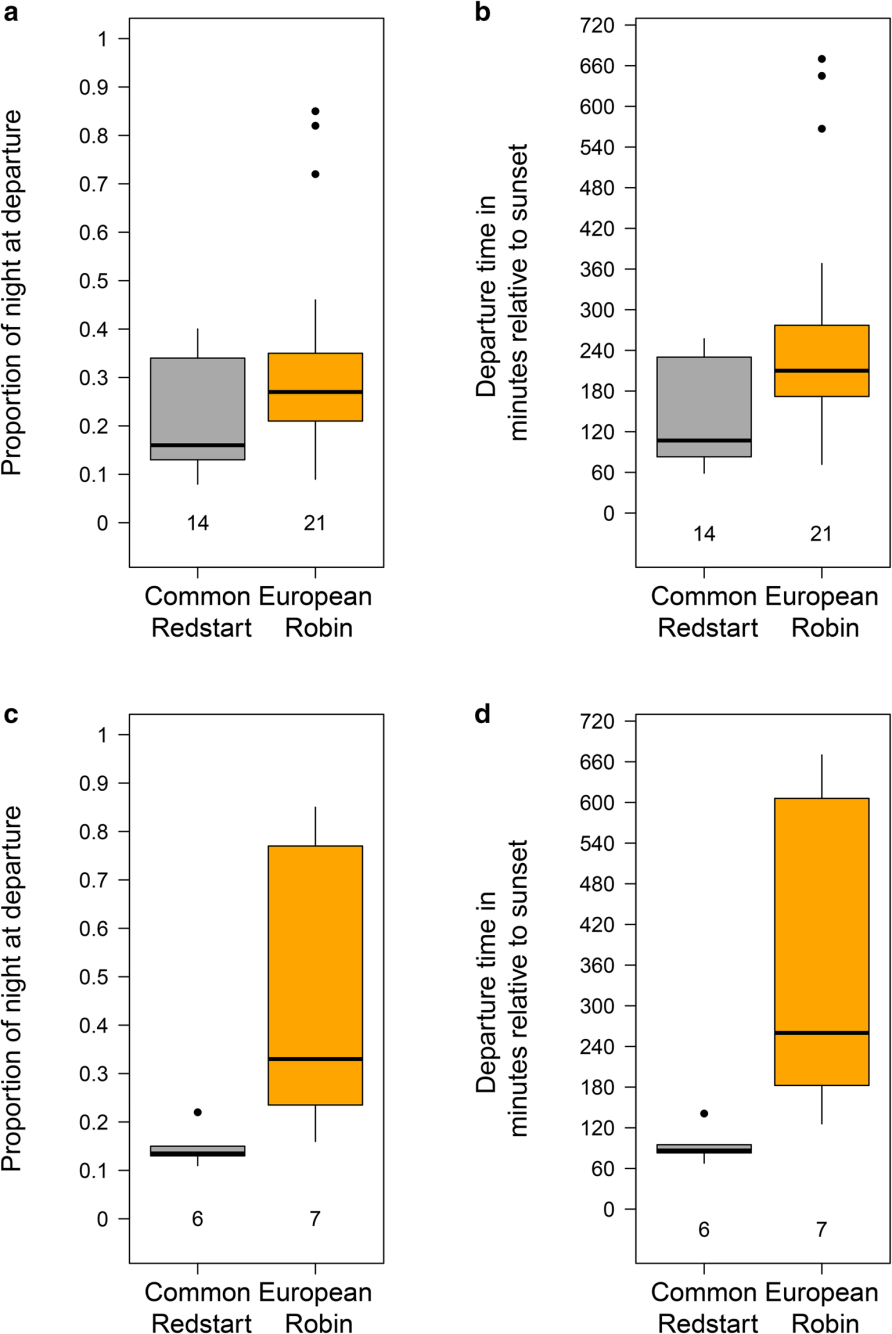
Variation in departure timing of common redstarts (grey) and European robins (orange) from Helgoland during autumn migration. Departure timing is given as (**a**, **c**) proportion of night at departure and (**b**, **d**) departure time in minutes after sunset for all birds (**a**, **b**) and for birds departing during the first night after capture only (**c**, **d**). Box plots show the 5th, 25th, 50th, 75th, 95th percentile and outliers (filled dots). Numbers at the bottom of the boxes represent sample sizes

**Fig. 5:**
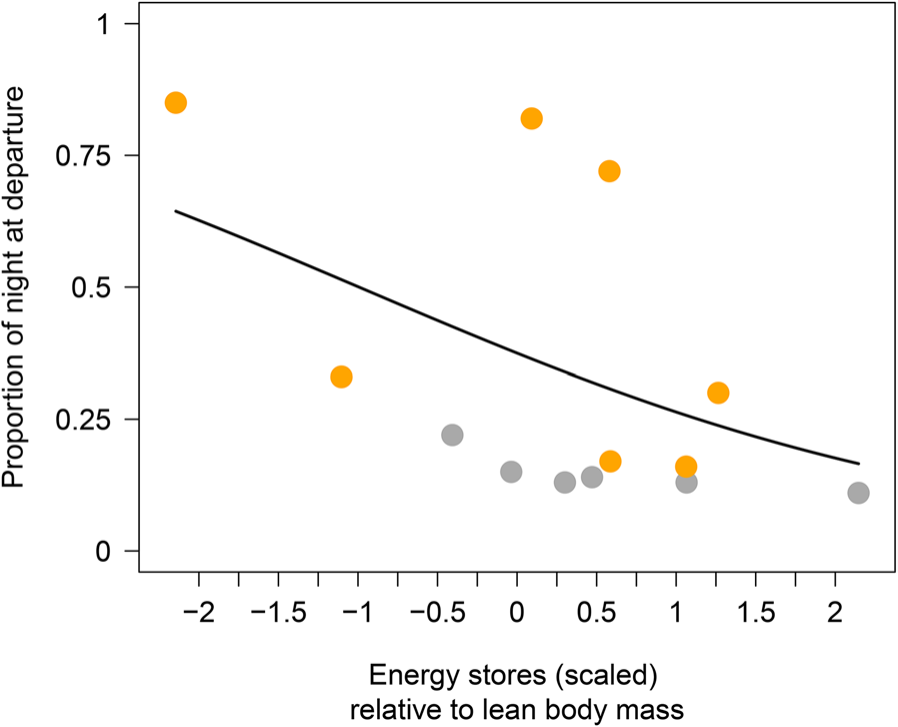
Effect of energy stores on nocturnal departure timing (proportion of night at departure) in common redstarts (grey, n = 6) and European robins (orange, n = 7) departing during the first night after capture. Depicted is the model estimate (solid black line) from the corresponding beta regression model, cf. Table 1b

All individuals of both species departed after sunset, with latest departures at the 40th percentile of the night in redstarts and the 85th percentile in the robins (Fig. 4a). Considering all departures, we found no significant differences in the nocturnal departure timing (proportion of night at departure) between the species (beta regression: intercept(redstart) = − 1.17 ± 0.22 (mean ± SE), species (robin) = 0.50 ± 0.27 (mean ± SE), p = 0.069; Fig. 4a). For the birds departing during the first night after capture, energy stores had a significant negative effect on the timing (proportion of night at departure), with relatively large energy stores co-occurring with relatively early departures (Table 1, Fig. 5) and redstarts set off significantly earlier than robins (Table 1, Fig. 4c). Regarding the actual departure timing in minutes after sunset, redstarts started their migratory flights significantly earlier than robins. This was true for all departures and when restricting departures to the first night after capture (Table 2, Fig. 4b, d). In the latter model, we found no general effect of the amount of energy stores on the departure timing (Table 2). When considering redstarts only, however, energy stores had a significant negative effect on the actual departure timing in minutes after sunset (LM: slope = − 1.33 min ± 0.41 min (mean ± SE), F_1,4_ = 10.6, n = 6, R^2^ = 0.73, p = 0.031).

**Table 1:** Beta regression models on nocturnal departure timing (proportion of night at departure) for (a) all birds, redstarts (n = 14) and robins (n = 21) and (b) birds departing during the first night after capture, redstarts (n = 6) and robins (n = 7)

Parameters	Estimate ± SE	z-value	p	pseudo-R^2^
(a) Model_all birds_				0.11
Intercept (redstarts)	− *1.17 ± 0.22*	− 5.27	< 0.0001	
Species (robins)	0.50 ± 0.27	1.82	0.069	
(b) Model_departing during the first night after capture_				0.58
Intercept (redstarts)	− *1.20 ± 0.37*	− 3.27	0.0011	
Species (robins)	*1.15 ± 0.46*	2.51	0.0120	
Energy stores (scaled)	− *0.45 ± 0.22*	− 2.07	0.039	

In the latter model, the effect of energy stores was included. Estimates, standard errors (SE), z-values and associated p-values of all parameters are shown. Significant effects are given in italics

**Table 2:** Normal linear regression models on species-specific difference in the nocturnal departure timing for (a) all birds, redstarts (n = 14) and robins (n = 21) and (b) birds departing during the first night after capture, redstarts (n = 6) and robins (n = 7)

Parameters	Estimate ± SE	t-value	p	R^2^	F
(a) Model_all birds_				0.21	8.9
Intercept (redstarts)	*2.090 ± 0.065*	32.00	< 0.0001		
Species (robins)	*0.251 ± 0.084*	2.98	0.0054		
(b) Model_departing during the first night after capture_				0.71	12.2
Intercept (redstarts)	*2.03 ± 0.099*	21.87	< 0.0001		
Species (robins)	*0.46 ± 0.135*	3.77	0.0037		
Energy stores (scaled)	− 0.12 ± 0.058	− 2.13	0.0591		

In both models, nocturnal departure timing was log10-transformed. Estimates, standard errors (SE), t-values and associated p-values of all parameters are shown. Significant effects are given in italics

**Fig. 6:**
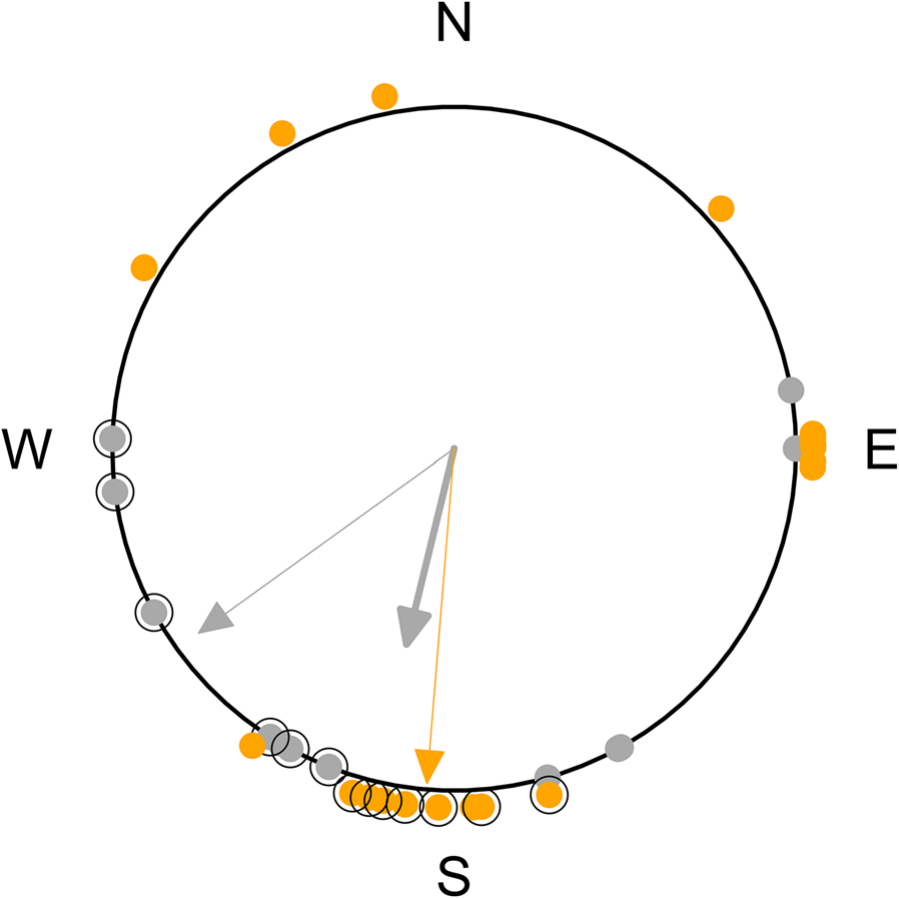
Departure directions of common redstarts (grey, n = 14) and European robins (orange, n = 21) from Helgoland during autumn migration. The six common redstarts and the seven European robins leaving Helgoland during the first night after capture are indicated by the black circles encompassing the corresponding filled dots. If departure directions were not uniformly distributed within a group and if the resultant mean vector length was significant, the mean direction of each group was represented by an arrow (thick arrow for all individuals of a species; thin arrow only for individuals departing during the first night after capture), whose length is drawn relative to the radius of the circle (= 1)

Departure directions of all redstarts were oriented (Rayleigh Test of Uniformity: ρ = 0.57, n = 12, p = 0.016) and showed a mean direction of 194°, whereas the directions of all robins did not depart from uniformity, i.e. they were not oriented (Rayleigh Test of Uniformity: ρ = 0.30, n = 20, p = 0.16; Fig. 6). For birds departing during the first night after capture, the departure directions of both species were oriented (Rayleigh Tests of Uniformity: redstarts, ρ = 0.89, n = 6, p = 0.003; robins, ρ = 0.98, n = 7, p < 0.0001; Fig. 6) and were not significantly affected by the energy stores (circular–linear correlations: p > 0.05). The mean direction of the redstarts was 232° and significantly different from the mean direction of 185° found in the robins (Watson’s two-sample test: T = 0.28, p < 0.01; Fig. 6).

## Discussion

Our quasi-experimental study shows that the decision-making process of migrant songbirds was in parts influenced by the variation in energy stores regarding the night-to-night departure probability (Fig. 3) and the nocturnal departure timing (Fig. 5). As such, our results are in agreement with correlative [18, 21–24], experimental [41, 43] and quasi-experimental studies [20] demonstrating that birds with higher energy stores are more likely to resume migration and do this earlier within the night than birds with lower energy stores (Tables 1, 2; Figs. 3, 5). In contrast to our expectations, we did not find differences in all departure decisions with respect to the two migratory strategies (long- vs- medium-distance migrants). However, redstarts set off earlier within the night than robins (Fig. 4b–d). This result supports former findings [18, 21, 24] and the hypothesis that species/populations with longer remaining migration distance start their nocturnal departure earlier within the night than species/populations with shorter remaining migration distance [9]. More quasi-experimental studies with higher sample sizes are required to get a better understanding of potential species-/migration strategy-specific differences in the decision-making process at a stopover.

### Night-to-night departure decision

The variation in energy stores was sufficiently large to significantly affect the night-to-night departure decision in the redstarts, with high energy stores inducing departure (Fig. 3) as expected from previous studies, reviewed in Schmaljohann and Eikenaar [7]. Although we found comparable variation in the energy stores of the robins (Fig. 1), the analogous pattern was absent in this species (Fig. 3) and other studies [51, 52]. This was unexpected since a recent study during autumn migration on Helgoland did find that high energy stores induced departure also in robins [18]. Potentially, this discrepancy is explained by the lower sample size (n = 21) and thus lower power of detecting an effect in our study in comparison with the 31 robins included in the study by Packmor et al. [18]. Furthermore, instead of resuming migration more robins might have left Helgoland to perform landscape movements [7, 53, 54] in search of a more suitable stopover site along the coast of the German Bight or even to reach their actual wintering range in the close vicinity [50, 55]. Since accomplishing such short-range movements requires only a relatively small amount of energy, the corresponding decision to depart from a stopover site is less strongly affected by the current energy amount than the decision to set off for a long-distance flight [41]. Thus, a relatively high proportion of robins departing on the first night after capture leaving Helgoland for a short-range flight will weaken the expected effect of variation in energy stores on departure probability.

According to the optimal migration theory, long-distance migrants are supposed to behave more like time-minimizers, while behaving more consistent with the energy-minimizing strategy is supposed to be favourable for medium-distance migrants [48, 49, 56]. It was, therefore, predicted and shown in at least two studies [18, 24] that long-distance migrants have a shorter minimum stopover duration than medium-distance migrants. The fact that this pattern was not present in our data is due to the five redstarts staying for more than 5 days (Fig. 2). Why they remained on the island so long is difficult to determine. In one individual, the initial energy stores at capture were only 0.01% of its lean body mass (Fig. 1) which might have prevented departure. The energy stores in the other four birds were 0.05 (two times), 0.11 and 0.15% of their lean body mass and therefore as high as or higher than the lowest minimum energy store of a departing redstart (Fig. 1). Potentially, these individuals may have experienced more favourable feeding conditions on Helgoland compared to the former stopover. This could be a signal in the decision-making process to remain at this more favourable stopover [41], as the exploitation of higher energy accumulation rates than before would be tantamount to an increase in the migration speed [48, 49, 56]. Alternatively, these long-staying redstarts might need longer to physiologically recover from the previous energetically demanding migratory flight than the others [5]. Regarding the potential influence of the weather on the departure decision, temperature, air pressure, precipitation and wind conditions (Additional file 1: Figure S1) did not deteriorate during the 5 days after the day of capture (Additional file 1). Therefore, it seems unlikely that the weather conditions strongly influenced the departure decision of the longer staying redstarts on Helgoland during the first few days.

### Departure decisions within the night

Regarding the nocturnal departure timing during the first night after capture, we found that birds with relatively high amounts of energy stores set off earlier within the night than leaner birds. High amounts of energy stores resulted in earlier take-offs (Fig. 5), a finding that supports earlier observations from correlative studies [21, 22, 24, 57]. Furthermore, redstarts set off significantly earlier during the first night after capture than robins (Fig. 4b–d). By starting the migratory flight shortly after sunset, birds may maximize their potential flight duration and the distance covered during that night, which in turn directly affects the overall speed of migration. It was therefore predicted [9] and has been recently shown [18, 21] that long-distance migrants depart earlier within the night than medium-distance migrants. Unexpectedly, this pattern disappeared when considering the departure timing, expressed as proportion of night at departure, of all birds (Fig. 4a).

In both species, departures from Helgoland during the first night after capture were oriented towards the seasonally appropriate direction according to the locations of ring recoveries regarding Helgoland (Fig. 6) [58]. The directions were not affected by the amount of energy stores. A potential explanation for the missing dependency might be that the sea crossings in our study were biased towards short distances during the first night after capture (n = 13, median = 48 km, first quantile = 45 km, third quantile = 65 km, range 45–520 km; after [22]). Hence, even little energy stores were sufficient to successfully reach the islands/mainland in most of these directions. The species-specific departure directions (Fig. 6) may be explained by the different general wind conditions experienced when leaving Helgoland, with easterly wind (circular mean: 86°) for the redstarts and westerly wind (circular mean: 255°) for the robins (Fig. 6, Additional file 1: Figure S1). In the six redstarts departing from Helgoland after the first night, wind direction correlated significantly with departure direction (circular correlation: p = 0.03). This, together with the higher variation in wind direction on these days (Rayleigh Test of Uniformity: ρ = 0.23, n = 6, p = 0.74) than on the night of capture (Rayleigh Test of Uniformity: ρ = 1, n = 6, p = 0), may explain the seasonally unexpected departure directions in the redstarts (Fig. 6; Additional file 2). Since the main wintering grounds of the redstarts lie in Africa south of the Sahara [59], they seemed to have allowed for drift [60], which is thought to be advantageous when being still relatively far from the migratory destination [61] and thus explains the stronger west component in the departure direction in comparison with the robins (Fig. 6). Departure directions in later nights were more scattered in both species. In contrast to the far distant wintering grounds of the redstarts in Africa, some robins passing Helgoland overwinter in Great Britain and northern Germany [50, 58] which may explain some of the westerly and easterly departure directions in the robin.

## Conclusion

Although we could not find supportive evidence for all of our hypotheses, we are convinced that quasi-experimental studies like ours and Goymann et al. [20] provide an important approach to study species-/migration strategy-specific differences in the migration behaviour and decision-making process in addition to correlative and experimental studies. The great advantage of studying many individuals of the same species at the same time is that the extrinsic conditions are “identical” for the birds. Hence, differences in their decision-making process are most likely to be strongly related to the between-individual differences of the intrinsic conditions and less to, among others, atmospheric conditions [62], predation pressure [63] or competition [64]. Relating detailed knowledge of the birds’ intrinsic condition, e.g. energy stores [7], age [65, 66], sex [67], migratory destination [21], physiology [5, 6] and immune system [68], to the departure decision may eventually allow formulating distinct conditions at which “most” individuals decide to resume migration or to remain at stopover. Such an approach neglects the migratory history of the birds before they entered the stopover site and their future expectations of better or worse conditions elsewhere [48, 69] which differ between individuals. Still, any in-between conditions may provide a good starting point for detailed experimental studies to investigate the relative importance of the different factors within the decision-making process at a stopover.

## Methods

### Study site and field procedures

**Fig. 7:**
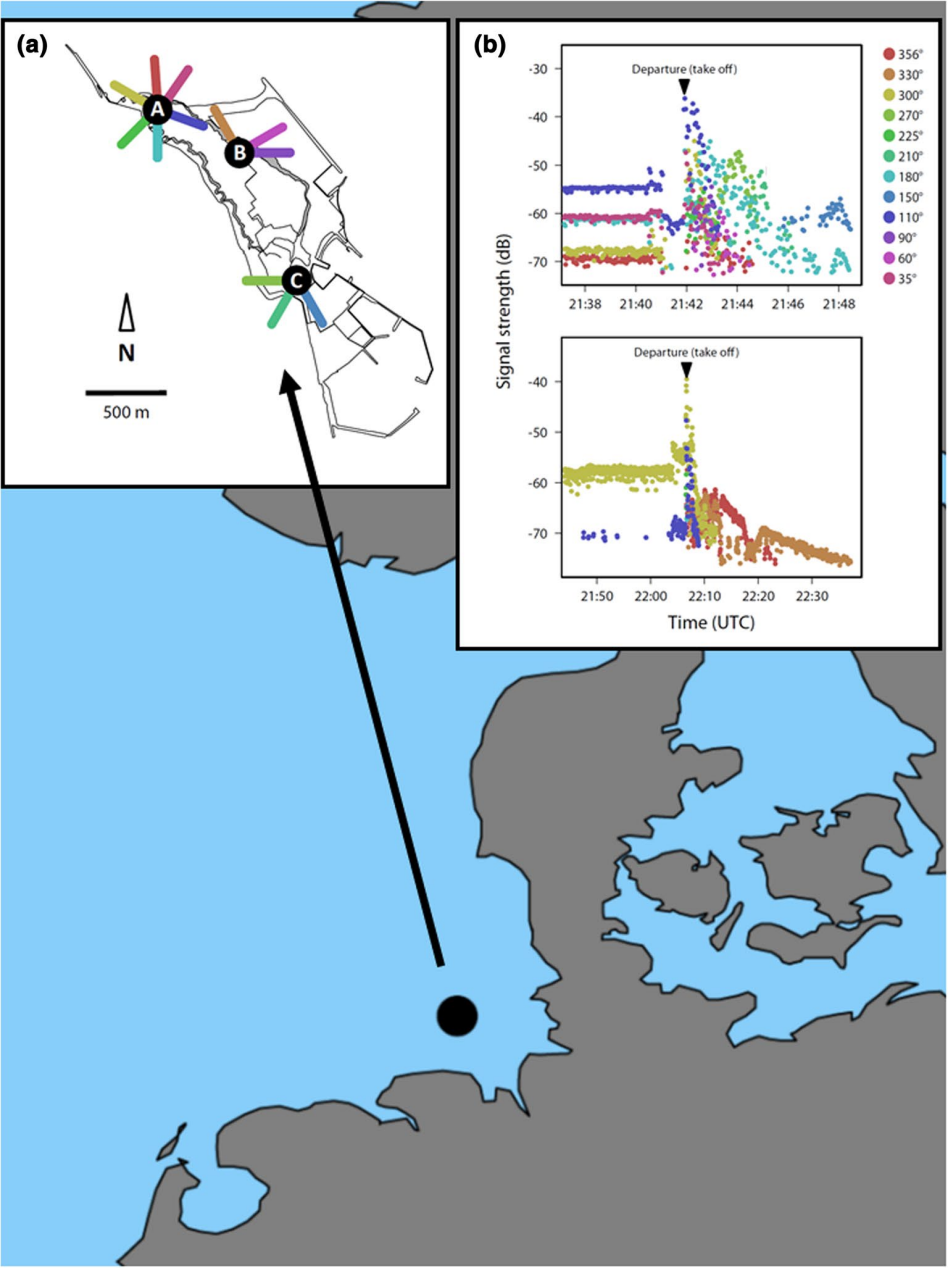
Location of the study site Helgoland in the German Bight. From there about 50 km are to be covered across the North Sea Bering Strait to reach the mainland. **a** The automated digital radio-telemetry system on Helgoland consists of twelve antennas at three sites (A, B, C). Coloured bars represent the different antennas and correspond to those given in (**b**). **b** Two nocturnal departure events as recorded by the system showing raw signal strength data over time (Coordinated Universal Time: UTC). The time of departure (take-off) defined as the time of highest signal strength and the estimated departure direction based on signals from the second half of the departure event are given. Colours denote signals received by different antennas aligned to directions given in the legend. This figure was created by using R, ver. 3.5.3 [76]

The study was conducted on Helgoland (54°11′N, 07°53′E), a small island (2 km^2^) in the North Sea about 50 km off the German coastline (Fig. 7). Redstarts and robins were trapped using mealworm-baited spring traps, funnel traps and/or mist nets. All redstarts were caught on the 2nd of September 2018 between 8 am and 1 pm (local time) and all robins on the 6th of October 2018 between 11.30 am and 3 pm (local time). For each bird we assessed: muscle size after Bairlein [70] on a scale from “0” (sternum sharp, muscles depressed) to “3” (sternum yet distinguishable, muscles slightly rounded), body mass (± 0.1 g) with an electronic balance and maximum wing length to the nearest 0.5 mm [71]. The latter was used as a measure of body size [72].

After ringing, each bird was fitted with a coded radio-tag (NTQB-2 Avian Nano Tag; 0.29 g; Lotek Wireless Inc., Newmarket, ON, Canada) using a leg-loop harness individually adjusted to the bird’s body size [73]. Mass of radio tags (including harness < 0.35 g) did not exceed 3% of the individual body mass of the species (min = 1.9%, max = 2.8%) [74]. All procedures were approved by the Ministry of Energy, Agriculture, the Environment, Nature and Digitalization, Schleswig–Holstein, Germany.

### Estimating energy stores

We estimated each bird’s energy stores following the approach detailed in Kelsey et al. [46]. First, we applied the species- and muscle score-specific equations to estimate the bird’s lean body mass.

for each redstart_i_ with a muscle score of “2”:

1 $$lean \;body\; mass_{{redstart_{i} , \;muscle\; score\; 2}} \;\left[ g \right]\; = \;6.69\; \left[ g \right]\; + \;0.08 \left[ {\frac{g}{mm}} \right]\;*\;wing\; length_{i} \;\left[ {mm} \right],$$

for each robin_i_ with a muscle score of “2”:

2 $$lean\; body\; mass_{{robin_{i} , \;muscle \;score \;2}} \;\left[ g \right]\; = \;2.48\; \left[ g \right]\; + \;0.17 \left[ {\frac{g}{mm}} \right]\;*\;wing \;length_{i} \;\left[ {mm} \right],$$

and for each robin_i_ with a muscle score of “3”:

3 $$lean \;body\; mass_{{robin_{i} ,\; muscle \;score \;3}} \left[ g \right]\; = \;2.77\;\left[ g \right]\; + \;0.17 \left[ {\frac{g}{mm}} \right]\;*\;wing \;length_{i} \;\left[ {mm} \right].$$

No individual had a muscle score of “0” or “1”. No redstart had a muscle score of “3”.

Second, we calculated the bird’s energy stores at capture based on the individual lean body mass as:

4 $$Evening\; energy\; stores_{i} \; = \frac{{\left( {body \;mass_{i} \; \left[ g \right] \;{-}\;lean \;body\;\; mass_{i} \; \left[ g \right]} \right)}}{{lean \;body \;mass_{i} \;\left[ g \right]}}.$$

### Recording of departure timing and direction

Departure timing and direction were determined using an automated digital telemetry system that, throughout the autumn migration season of 2018, continuously recorded signals on the used frequency (150.1 MHz) which is free for animal tracking in Germany (Federal Network Agency). Our telemetry system consists of four radio receiving towers at three sites on Helgoland (Fig. 7), each equipped with a SensorGnome receiver (http://www.sensorgnome.org) and three antennas (6EL Yagi antennas; Vårgårda Radio AB, Sweden), for further details see Müller et al. [21]. This system is part of the Motus Wildlife Tracking System, see https://www.motus.org and Taylor et al. [75]. The overall array of the 12 horizontally mounted antennas was aligned radially at intervals of 30° (Fig. 7). Departures of birds as obtained by the system are generally characterized by a rapid increase in signal strength detected from all/most antennas (a bird is setting off the ground), followed by a decline in signal strength from a decreasing number of antennas until the loss of signal (a bird is leaving the site in a specific direction and vanishing out of the survey volume) (Fig. 7). Individual take-off time is characterized by the time of the highest signal strength during each departure event (Fig. 7) and automatically determined for each bird in the same way by a standard algorithm. Based on the take-off time, the algorithm calculated the respective temporal difference between initial capture and departure (minimum stopover duration in days), the bird’s nocturnal departure timing in relation to night length (the proportion of night at departure because night length changes with season) and its departure time in minutes after sunset. The algorithm further automatically rated the departure direction of the birds by calculating a weighted circular mean of the directions the receiving antennas were aligned to. It excludes signals from the first half of the departure event to reduce the chance of taking misleading detections from antennas’ back and side lobes into account. Directions of signals included in the circular mean were weighted by their temporal proximity to the last detection. Whenever pivotal antennas (antennas aligned to a direction close to the calculated departure direction) failed to record signals during the departure event and/or the signal got lost shortly after a birds’ take-off (< 3 min), the obtained departure directions were discarded, as these were probably imprecise. As a result, the specific take-off pattern was missing for five of the 40 radio-tagged birds in total. As we could not ascertain their departure times, these birds were excluded from the study. We further omitted the departure direction for two redstarts and one robin because the tracked departure event was shorter than 3 min and thus not trustworthy. The automated digital telemetry system provided precise and observer unbiased identification of the birds’ minimum stopover duration, departure timing within the night and departure direction, for further details see Müller et al. [21].

### Weather data

Meteorological data were obtained from an automated weather station operated by the German Meteorological Office on Helgoland (DWD; https://opendata.dwd.de/climate_environment/CDC/observations_germany/climate/hourly/). Out of its hourly measurements, we used wind speed [m/s], wind direction [°], precipitation [mm] and air temperature [°C] to characterize the weather conditions the birds experienced at sunset (nearest full hour) on their day of capture and for each following day until departure (Additional file 1: Figure S1).

### Statistical analyses

The statistical analyses were implemented using R, ver. 3.5.3 [76]. To assess whether the departure decisions are correlated with variation in the energy stores of the species and whether there are species-specific differences in these decisions, we fitted normal linear regression models or beta regression models, the latter by using the “betareg” function implemented in the “betareg” R package [77]. Beta regression models were applied to model variation in nocturnal departure timing, here as the proportion of night at departure. Assessing the effect of variation in energy stores on the departure decision was restricted to birds leaving Helgoland during the night after capture because energy stores change during a stopover [7]. When the residual analyses violated the model’s assumptions and when the transformation of variables did not sufficiently mitigate these violations, we applied non-parametric tests. All data used in this study are presented in Additional file 2: Table S2.

To analyse departure directions, we applied circular statistics using functions of the R packages “CircStats” [78] and “circular” [79]. For each circular data set, directions were tested for uniformity with the Rayleigh test of uniformity [80, 81]. A significant result indicates that circular data do not follow a circular uniform distribution. Only for such data sets, we calculated the circular mean and the mean resultant length as a measure for concentration [82]. Circular–linear correlations were calculated following the methods described by Jammalamadaka and SenGupta [80]. The p-value for a circular–linear correlation was approximated by a randomization test. For each circular and linear variable, random samples with replacement were drawn and the circular–linear correlation coefficient of these values was estimated. We used 10,000 random replications in each case. The number of replicates with a correlation coefficient larger than the correlation coefficient associated with the original data set, divided by the total number of replications, provides a robust estimate of the corresponding p-value [83].

## Supplementary information

**Additional file 1: Figure S1.** Weather data of 5 days after capture or common redstarts.

**Additional file 2: Table S1.** Raw data of the study. Time: local time; Species: CR = common redstart, ER = European robin; Muscle: muscle score; Wing: wing length in mm; Bodymass: body mass in g; take.off: local time of take off; dep.dir: direction at departure; days.on.island: days spent on the island; dep.min.sunset: departure in min after sunset; dep.realtive.night: departure relative to night length; wind.speed: wind speed in m/s at departure; wind.dir: wind direction at departure.

## Data Availability

All data generated or analysed during this study are included in this published article and its additional files.

## Abbreviations

LM: Linear model
SE: Standard error
