# Supplementary material for: A quasi-experimental approach using telemetry to assess migration-strategy-specific differences in the decision-making processes at stopover
Source: BMC Ecol. 2020 Jul 8;20:36. doi: 10.1186/s12898-020-00307-5 (PMC7346510; doi:10.1186/s12898-020-00307-5)
Supplement: Supplementary file 1 — Additional file 1: Figure S1. Weather data of 5 days after capture or common redstarts. [file 12898_2020_307_MOESM1_ESM.docx]

**A quasi-experimental approach using telemetry to assess migration-strategy specific differences in the decision-making processes at stopover**

Heiko Schmaljohann^1,2,$,^* and Thomas Klinner^2,^*

^1^Institute for Biology und Environmental Sciences (IBU), Carl von Ossietzky University of Oldenburg, Carl-von-Ossietzky-Straße 9-11, D-26129 Oldenburg, Germany

^2^Institute of Avian Research, An der Vogelwarte 21, 26386 Wilhelmshaven, Germany

$ author of correspondence: heiko.schmaljohann@uol.de

* These authors contributed equally to this work.

Content

Figure S1. Air temperature, rain, wind speed and wind direction on Helgoland.


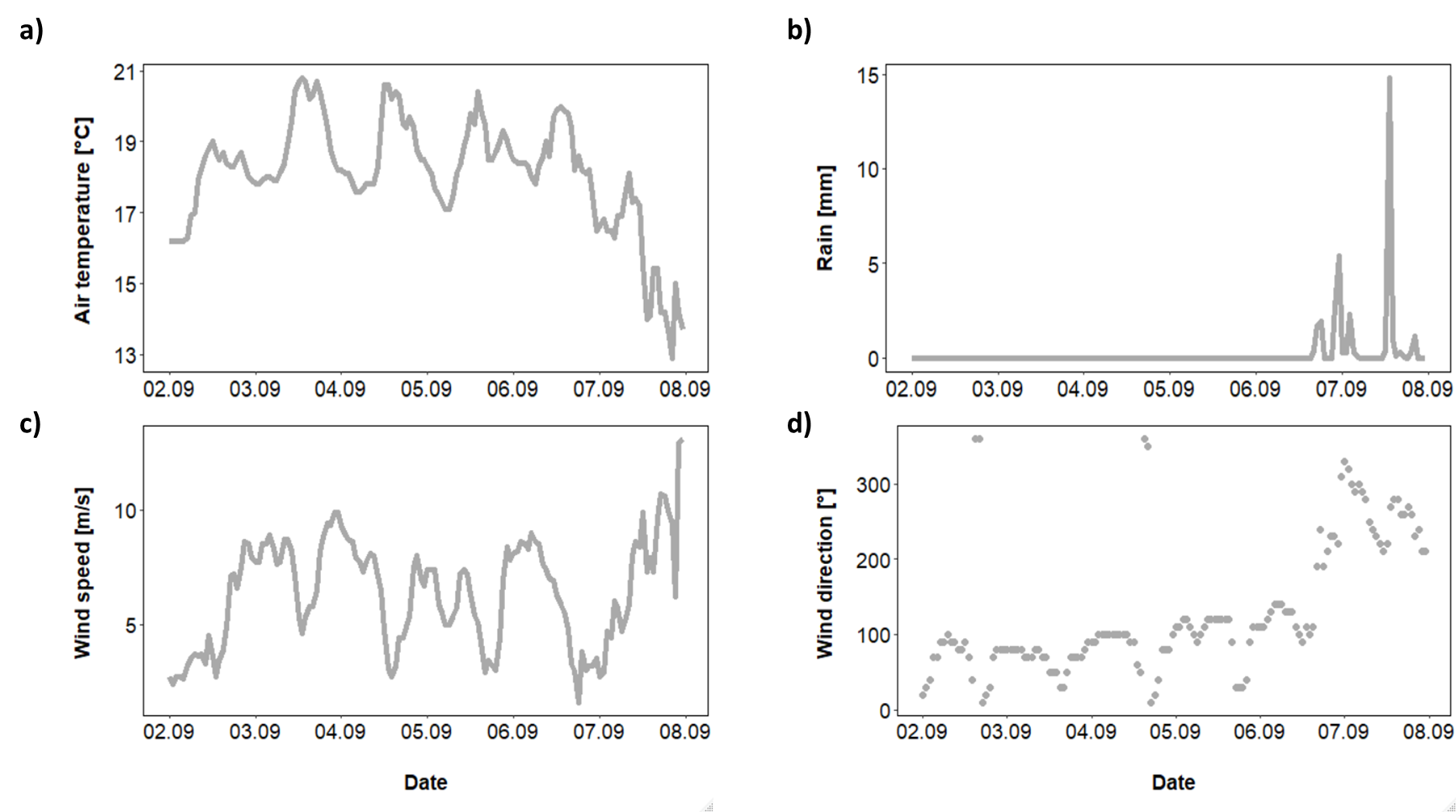


**Figure S1** Air temperature, rain, wind speed and wind direction on Helgoland at the beginning of September in 2018. Hourly data were obtained from an automated weather station operated by the German Meteorological Office on Helgoland (DWD; https://opendata.dwd.de/climate_environment/CDC/observations_germany/climate/hourly/).
